# Supplementary material for: Can athletes be tough yet compassionate to themselves? Practical implications for NCAA mental health best practice no. 4
Source: PLoS One. 2020 Dec 31;15(12):e0244579. doi: 10.1371/journal.pone.0244579 (PMC7774983; doi:10.1371/journal.pone.0244579)
Supplement: Attachment — Submitted filename: Point-by-point answer to Reviewers_FINAL.docx [file pone.0244579.s001.docx]

Point-by-point response to (Editor and) Reviewers

| Comment | Response |
| --- | --- |
| Editor: Policy discussion component | The authors:   1. Replaced the words “recommend” and “recommendation” throughout the manuscript with “suggest”, “suggestion” or “practical implication”:   Lines: 42, 100, 418, 420, 429, 432, 453, 456, 467, 473, 478, and 544 and   2. Used softer language, such as “may be” instead of “is” (line 419), “indicate” instead of “suggest” (lines 427 and 432), and “may support” instead of “support” (line 450). |
| Editor: PLOS ONE's style requirements | The authors checked the manuscript, so it meets the journal’s requirements. |
| Editor: Please consider whether individual events like those listed in the introduction are necessary to your presentation | The individual events in the Introduction were deemed necessary. Therefore, they were included in the revised version of the manuscript, as well.  However, and concerning the timeline, changes have been made in the beginning of the “NCAA and Mental Health Best Practice” section, so the flow is better. Except for adding a whole new first paragraph, the following phase was added (lines 81-2): “…triggered by the recent catastrophic events presented above…” |
| R1: “The findings indicate (but sometimes merely suggest) that the measures are  complementary and were associated with aspects of mental health.” | The authors changed wording from “suggesting” to “indicate” in Discussion: “In other words, the main effects indicate both can support MH.” Line 427. |
| R1: “…the authors fail to cite the rich historical and empirical basis for  the role of mental health athletes and its impact athletic performance which  would buttress the general principle behind the study. This is important because for many years the field of sport psychology discounted the role of mental health in athletes.” | Major changes have been made in the beginning of the “NCAA and Mental Health Best Practice” section. The relationship between mental health and athletic performance has been addressed by a whole new first paragraph (lines 67-73). |
| R1: Major concerns regarding this include the fact there has been an continues to be a lack of consensus as to 1) exactly what the construct of Mental Toughness is, 2) whether it is unidimensional or consists of multiple factors and, 3) whether sport-specific measures are more appropriate than broader (i.e., measures designed for all athletes or the general population). | The comment is 100% accurate. However, the scope of this manuscript is not to offer an extensive literature review on MT. The authors added a sentence including a chapter from a 2020 book, to which readers, who would like to investigate deeper, could refer (lines 175-77). |
| R1: Why focusing on these two constructs at the exclusion of other relevant and validated measures that also have been found to be associated with positive mental health benefits? Have these measures been neglected? Have they been found to be more useful in related research? Has no one used them in tandem as you did in your study? You provided some information that supports the link of these measures but make no attempt at all to provide either theoretical or empirical evidence that these measures are more central to general mental health than other constructs. | These are important questions, but they are largely addressed in the following section, Mental Toughness and Self-Compassion. In addition, SC may not be more central to MH in student-athletes. However, is that a prerequisite for investigation? An abundance of research shows positive connections between MT and MH and SC and MH, but a dearth of research investigates these two constructs together. Qualitative research shows SC and MT can work together to support MH, but that link has not yet been established quantitatively.  The following edits and additions were made in response to this comment: “As is elaborated below, though an abundance of studies have investigated the positive mental health benefits of these  two constructs separately, few studies have considered them in tandem. Qualitative research supports a collaborative relationship between MT and SC (e.g., Wilson et al., 2019), but quantitative studies are still needed for corroboration. This study helps clarify, philosophically speaking, if there is justification for viewing these two constructs as complementary, rather than competing approaches to wellness.”  (lines 101-8)  We also added these two sentences to the SC section to strengthen the rationale for investigating SC: “While many constructs link to MH, research on the key factors of therapeutic action, suggest SC is essential (Galili-Weinstock et al., 2018). In the case of reducing depressive symptoms, an experimental study (Diedrich et al., 2014) showed SC was more effective at regulating negative emotions than cognitive appraisal or acceptance conditions.” (lines 134-7) |
| R1: For, example, hypothesis three in which you predict that D-I athletes will score higher in your mental health measures could (should) be traced back to the mental health model long ago posited by Morgan. This context would help deepen the theoretical basis for your study. | We added a sentence: “However, according to Morgan’s mental health model (the Iceberg Profile), athletes of higher levels of performance (e.g., NCAA Division I programs) exhibit greater positive mental health than those of lower levels (e.g., NCAA Division III programs).”(lines 116-19). |
| R1: Are there in fact studies, theories, or perspectives that have been expressed in the literature suggesting or contending that MT and SC are competing, incongruent or orthogonal? | The only study that has investigated both constructs in the sporting context is the Wilson et al., (2019), which is mentioned in the manuscript. In fact, some of their results have been used to support the rationale of our study.  There are no specific theories to suggest that these two constructs are competing. That does not necessarily prove that MT and CS are competing, incongruent, or orthogonal. What we do know for sure is that there is a gap in the literature, which we are trying to start covering by providing some evidence (which could lead to more investigation and eventually to a creation of a theory). In addition, one of the goals of the “Mental Toughness and Self-Compassion” section was to provide evidence as to why it would worth investigating these two constructs together in the sporting context and, especially, through the lens of mental health. |
| R1: It might be pointed out that Loehr’s measure of MT suffers from serious validity issues, a problem not uncommon with sport-specific inventories. | There is indeed criticism concerning PPI’s psychometric soundness. However, Loehr’s name at that section of the manuscript was used because he made the term popular in the ’80. The readers, who would like to know more MT instruments, will have to see the 2020 Gucciardi chapter we mentioned above. (lines 175-77). |
| R1: This is the crux of the problem with MT, aside from the decidedly generic definitions of MT used in the literature, no one who studies it agrees on what precise the construct is. The debate goes beyond whether MT is unidimensional or has several factors. In fact, Gucciardi’s initial MT scale (the AFMTI Gucciardi, 2009) was composed of four sub-factors. Among researchers who continue to conceptualize MT as multifactorial, there is a lack of consensus as to both the number of sub-factors (they range from two to 12 among the six most commonly used measures of MT) or what those factors are. what those factors are or even the number of factors. Other researchers contend that MT measures should be specific to the target population; hence the development of sport-specific MT questionnaires. There is also the contention that MT shares considerable overlap with resilience, a construct of considerable currency in sport psychology research. None of this is to say that you either selected the wrong construct or wrong measure of a construct to employ in your study. But you have neglected to point out the issues surrounding the construct of MT fully as the perspectives voiced by critics of MT research. As stated by Jones (2002): “Mental toughness is probably one of the most used but least understood terms used in sport psychology.” | Concerning the issues that R1 points out, indeed, there is significant ambiguity in the MT literature. However, the aim of this paper is not to fully address these issues.  As mentioned above, the authors addressed the Reviewer’s concerns by adding a sentence including a chapter from a 2020 book, to which readers, who would like to investigate deeper, could refer (lines 175-77). |
| R1: You need to provide citations that support the contention that Gucciardi’s more recent definition of MT has been gaining acceptance. | A reference was added: Anantasagar, T., Abbott, K., Stamatis, A., & Papadakis, Z. (2018). Mental toughness in sport: Moving towards conceptual clarity and consensus. In International Journal of Exercise Science: Conference Proceedings (Vol. 2, No. 10, p. 81). Line: 172 |
| R1: Are these correlations consistent across all MT measures? Have sex differences been consistently found across all MT measures? I would guess not. Also, the fact that the evidence you cite that MT scores are higher in male athletes is ‘preliminary’ implies that 1) MT measures do not have published norms and 2) early studies did not find sex differences. | Indeed, all these correlations have not been confirmed by all instruments that claim to measure MT. However, the results we have so far have not been inconsistent either.  In other words, several instruments, such as AfMTI, MTI, MTQ18 have been used to collect data that provide evidence for those correlations we have included in our manuscript. There is no evidence from other MT instruments that is not in accordance with those results. Therefore, the current state of evidence was deemed enough to support our rationale and hypotheses. The above applies on sex differences, as well. |
| R1: Is this a consistent finding? Have others replicated this? Are the findings based on a particular MT measure? Is this the only study that has found a link with a negative outcome in athletes? | The findings on rehabilitation have not been replicated. To the best of our knowledge, there is one study (see below) that showed no significant main effects of positive or negative mental toughness on injury outcome. However, that study is not on rehabilitation. The Levy paper has been a “classic” reference concerning the “dark” side of MT.  Petrie, T. A., Deiters, J., & Harmison, R. J. (2014). Mental toughness, social support, and athletic identity: Moderators of the life stress–injury relationship in collegiate football players. *Sport, Exercise, and Performance Psychology*, *3*(1), 13. |
| Based on points made earlier, you should elaborate on your rationale for choosing this scale to the exclusion of the six or so others widely used in sport research. Moreover there is an argument that sport-specific scales, in some cases, are not necessarily more efficacious than general measures that have proven construct validity and have been shown to be effective in a wide range of situations and populations (e.g., anxiety measures). Provide enough evidence that your reader doesn’t second guess your decision. | The comments are accurate. There is a plethora of MT instruments. For instance, there are at least 12 instruments that have been developed to measure MT in sport only (see below in chronological order): Instrument (with acronym) \| Author(s) \| Year; Psychological Performance Inventory (PPI) \| Loehr \| 1986; Mental Toughness Questionnaire-48 (MTQ-48) \| Clough, Earle, and Sewell \| 2002; Mental Toughness Questionnaire-18 (MTQ-18) \| Clough, Earle, and Sewell \| 2002; Mental Toughness Inventory (MTI) \| Middleton, Marsh, Martin, Richards, and Perry \| 2005; Psychological Performance Inventory-Alternative (PPI-A) \| Golby, Sheard, and van Wersch \| 2007; Mental, Emotional, and Bodily Toughness Inventory (MeB-Tough) \| Mack and Ragan \| 2008; Australian Football Mental Toughness Inventory (AfMTI) \| Gucciardi, Gordon, and Dimmock \| 2009; Cricket Mental Toughness Inventory (CMTI) \| Gucciardi and Gordon \| 2009; Sport Mental Toughness Questionnaire (SMTQ) \| Sheard, Golby, and van Wersch \| 2009; Mental Toughness Scale (MTS) \| Madrigal, Hamill, and Gill \| 2013; Mental Toughness Index (MTI) \| Gucciardi, Hanton, Gordon, Mallett, and Temby \| 2015; Mental Toughness Questionnaire-10 (MTQ-10) \| Papageorgiou, Malanchini, Denovan, Clough, Shakeshaft, Schofield, and Kovas \| 2018 Not only it is not the purpose of this study to comment on the psychometric soundness of all MT instruments (whether developed based on the specific or the global view), but it would also be inefficient in terms of space. Therefore, instead of providing evidence on why we did not choose any of the other measurement tools, we provided evidence on the reliability of scores and the validity of the inferences of those scores of the instrument we chose to use (i.e., MTI) (lines 255-264). In addition, at the very end of the manuscript, we explicitly state that there are other psychometrically-sound instruments out there for all three constructs (lines 588-589). Lastly, for readers who are interested in psychometrics, the 2020 Gucciardi chapter should provide enough evidence and a large amount of references for additional research (lines 177). |
| R1: This statement seems like wishful thinking: the interaction is “buffering” yet not “statistically significant” but it still “warrants further elaboration”. It is perfectly acceptable to suggest that further research is needed, but you shouldn’t imply you found something in the absence of statistical significance. Needs clarification. | We added clarification to this section: “… in this sample…” and “So, chance observation of the buffering relationship cannot be ruled out. “ See lines 445-6. |
| R1: “SC seems to facilitate the effective use of MT” You need to be clear here as to whether this is based on statistically significant findings or not. Needs clarification. | We added a clause to lines 449-450 to make it clear that the direct and indirect effects were  statistically significant: “…via statistically-significant direct and indirect effects (i.e., partial mediation).” |
| R1: This sounds an awful lot like a model of resiliency; the idea that it either increases or decreases (granted, the model also allows for no change) after experiencing adversity. | The Reviewer is definitely right to detect a lot of similarities. However, MT and resilience differ conceptually in terms of entity (MT applies on people only vs. resilience applies on systems/groups/communities, including people) and conceptual theme (MT applies on psychological factors only vs. resilience applies on a broader range of protective elements, such as economic infrastructure). In short, resiliency is conceptually broader than MT. |
| R2: On Pg 4, Line 83, What is a MH environment? This is not the language used by the NCAA and is not accurate. | Indeed, this is not NCAA language. Therefore, we changed “… that promote MH environments…” to “…that promote student-athletes’ MH issues management…” (Line 90) |
| R2: Pg 7 Line 165—167. How is MT interrelated to each of those concepts? Depression and thriving? This needs more explanation. | Great comment! Addressed by adding positively or negatively in all these interrelations (lines 182-3). |
| R2: Why was it hypothesize that DI athletes would score higher in MT, SC, and MH? There doesn’t seem to be any prior evidence to support this hypothesis. Clarify. | This comment was addressed in conjunction with a comment from R1. There is some preliminary evidence (Stamatis et al., 2018) and some theory (e.g., MHM) that deepens the theoretical basis for our study. Combined, this was deemed enough to develop and test Hypothesis #3.  In more detail, we added a sentence “However, according to Morgan’s mental health model (the Iceberg Profile), athletes of higher levels of performance (e.g., NCAA Division I programs) exhibit greater positive mental health than those of lower levels (e.g., NCAA Division III programs)” and reconstructed that whole paragraph (lines 115-19). |
| R2: Much of the discussion focuses on how the results found that mental toughness is  related to the mindfulness subscale of SC (.45), but there is no mention of the  relationship to self-kindness (.42) which is nearly as strong. What explanation do the authors have for this relationship? | Thank you for this keen observation. We hope the following provides some insight.  Specific comment: While nearly as strong of a positive relationship (.42) was found between kindness and MT, this connection is more perplexing. Though research continues to clarify the central conceptual attributes of MT, there are no obvious ways to explain why kindness bridges MT and SC. One theoretical possibility is that kindness links MT and SC via an ability to appraise challenging situations without descending into destructive self-criticism. Kindness is a way of appraising personal limitations as opportunities to focus on support and nurture the self by asking “What do I need to care for myself in this moment?” (Germer & Neff, 2019). MT appraises challenges as opportunities to thrive by blocking out negative thoughts and directing attention away from unhelpful distractions (Weinberg et al., 2017). In this way, we theorize that appraisal may be tough in its disciplined focus, yet kind when facing personal limitations. The authors conclude with limitations and recommendation for future studies. See lines 548-553.  General comment: Yes, we did explore the relationship between MT and the other sub-scales of SC. However, let’s not forget that -based on the Wilson et al., 2019 study- one of our objectives was to confirm if mindfulness underpins the MT-SC relationship. That’s one of the main reasons we are focusing on the Discussion on the MT/mindfulness results.  In order to avoid further confusion, we changed the title of the manuscript by substituting the word” kind” to “compassionate”. |
| R2: Was there an opportunity for participants to identify as a gender other than male or female? If not, how might that have affected the validity of the study? | Great comment!  We added a sentence in the Future Studies section: “In relation to that, and since the current study did not provide an opportunity for participants to identify as a gender other than male or female (delimitation), future designs should try to avoid gender bias, since that may lead to the lack of incorporation of gender data into evidence-based practice.” (Lines 582-586) |
| R2: “…SC seems to facilitate the effective (remove effective) use MT.” This doesn’t make sense. There is no such thing as an effective (or ineffective) use of mental toughness. Overall, some of the conclusions drawn about the relationship between MT and SC are too broad considering this study only found a moderate correlation between the two constructs. Some of these implications should be dialed back. | In this section, we linked our finding to the Wilson article, using it to provide a possible way of understanding how MT and SC might collaborate. We hoped that by 1) linking the implications to research by Wilson and 2) openly suggesting that we were theorizing, the implications would be seen as food for thought and for future investigation. |
| R2: This section would benefit from discussion of evolving gender norms. There are now many strong, female athlete role models that defy these norms, as well as more outspoken male athletes who have shared their struggles with mental health (e.g., Michael Phelps). | Excellent point. Thank you for this insight. We incorporated it into the following sentence.  “Additionally, although gender norms are evolving and becoming less rigid (e.g., Michael Phelps disclosure of struggles with depressive symptoms), they create a kind of straitjacket that restrict behaviors beyond the norm.” Lines 499-500 |
| R2: These recommendations improve best practices by providing specific practical psychological steps for athletes, coaches, and stakeholders invested in the collective task of more fully realizing best practice No. 4.” The recommendations are not actually very specific. It would be helpful to provide more information on how to enhance MT and SC within student athletes. | Agreed, thank you for this suggestion. As noted, the methods/findings of this study limit what we can recommend. However, we added the following about next steps involving a training program that might be applied to student athletes.  “Building on these recommendations will involve identifying specific training methods and techniques for teaching MT and SC to student-athletes and coaches. One promising place to start with regard to SC is to adapt and observe the effects of the Mindful Self-Compassion Program—an eight-week, 24-hour training that involves guided meditation and discussion sessions—on student-athletes (Germer & Neff, 2019). If an equivalent program for MT were developed, that too might be applied and evaluated.” Lines 548-553 |
| R2: I’m curious why there is not mention of sport psychologists or sport psychology consultants as they are the ones most likely to implement MT, SC, and/or mindfulness training? | Great comment!  We edited the following sentence by adding the parenthesis on line 527: “MH clinicians and practitioners (e.g., sport psychologists, sport psychology consultants)…” |
